# Multi-omics analysis of functional substances and expression verification in cashmere fineness

**DOI:** 10.1186/s12864-023-09825-0

**Published:** 2023-11-28

**Authors:** Yanan Xu, Yu Zhang, Yuting Qin, Ming Gu, Rui Chen, Yinggang Sun, Yanzhi Wu, Qian Li, Yanjun Qiao, Xiaowei Wang, Qiu Zhang, Lingchao Kong, Shuaitong Li, Zeying Wang

**Affiliations:** College of Animal Science & Veterinary Medicine, Shenyang Agricultural University, Shenyang, 110866 China

**Keywords:** Liaoning cashmere goat, Cashmere fineness, Transcriptomics, Translatomics, Proteomics, Metabolomics

## Abstract

**Background:**

Numerous factors influence the growth and development of cashmere. Existing research on cashmere has predominantly emphasized a single omics level. Integrating multi-omics analyses can offer a more comprehensive understanding by encompassing the entire spectrum. This study more accurately and comprehensively identified the key factors influencing cashmere fineness using multi-omics analysis.

**Methods:**

This study used skin tissues of coarse cashmere type (CT_LCG) and fine cashmere type Liaoning cashmere goats (FT_LCG) for the analysis. This study employed an integrated approach involving transcriptomics, translatomics, proteomics, and metabolomics to identify substances associated with cashmere fineness. The findings were validated using parallel reaction monitoring (PRM) and multiple reaction monitoring (MRM) techniques.

**Results:**

The GO functional enrichment analysis identified three common terms: multicellular organismal process, immune system process, and extracellular region. Furthermore, the KEGG enrichment analysis uncovered the involvement of the arachidonic acid metabolic pathway. Protein expression trends were verified using PRM technology. The expression trends of KRT79, as confirmed by PRM, were consistent with those observed in TMT proteomics and exhibited a positive regulatory effect on cashmere fineness. Metabolite expression trends were confirmed using MRM technology. The expression trends of 9 out of 15 validated metabolites were in agreement with those identified in the non-targeted metabolomics analysis.

**Conclusions:**

This study employed multi-omics analysis to identify key regulators of cashmere fineness, including *PLA2G12A*, KRT79, and prostaglandin B2. The findings of this study offer valuable data and establish a theoretical foundation for conducting comprehensive investigations into the molecular regulatory mechanisms and functional aspects of cashmere fineness.

**Supplementary Information:**

The online version contains supplementary material available at 10.1186/s12864-023-09825-0.

## Background

The cashmere goat, renowned for its significant economic value, has become increasingly popular daily. With the growing demand for cashmere, expectations regarding its quality have also increased. Cashmere fineness is a pivotal economic indicator and a cornerstone for selection and breeding endeavors [1]. It is widely recognized that finer cashmere possesses greater economic value and results in superior textile products. Nonetheless, the prioritization of high cashmere yield has sometimes led to the neglect of fineness and quality control, resulting in a gradual increase in cashmere fineness over time. This concern has prompted researchers to direct their efforts toward decreasing cashmere fineness in the Liaoning cashmere goat (LCG), a vital genetic resource in China celebrated for its abundant cashmere yield [2]. However, its cashmere fineness remains relatively coarse.

Cashmere fiber growth is a complex physiological process influenced by many factors, with genetic factors playing a particularly significant role. Several studies have implicated genes like *CES4A, COCH, ADIG, CCL24, PDE8B, PLIN2, HSPB7, K38C3, CASP1*, and *PRLR* in the regulation of cashmere growth and fiber characteristics, including fineness [3]. Association analysis of blood DNA and cashmere production performance in LCG indicated potential associations between genes such as *KRT26, TCHH, COL1A1, PRL, MSTN, IGFBP-3, INHA, PGR*, and *RARG*, and cashmere fineness, suggesting their utility as molecular markers for LCG breeding [4–8]. Keratin, a multifunctional protein involved in various cellular processes, plays a direct role in hair follicle development and functionality [9]. Studies have shown that genes such as *KAP6.1, KAP7.1, KAP8.1, KAP8.2*, and *KAP11.1* exhibit lower expression levels in primary hair follicles than secondary hair follicles, demonstrating a significant correlation with cashmere fineness and their potential pivotal role in regulating cashmere fiber diameter [10–12]. Furthermore, minor alterations in gene function ultimately manifest at the metabolic level during the cell’s life activities. Research has demonstrated that metabolites such as Gly-Phe and taurine deoxycholate can influence cashmere growth, development, and fineness [13]. Nevertheless, most studies have concentrated on individual omics analyses, neglecting the combined interplay of genes, proteins, and metabolites in cellular processes. The essential factors governing cashmere fineness warrant further comprehensive investigation.

Advancements in high-throughput sequencing technology have led to an increased application of omics techniques in studying cashmere-related traits. Guo et al. demonstrated the feasibility of multi-omics analysis by examining metabolite and protein expression profiles in the rumen tissue of goats subjected to varying diets. Their findings shed light on the adaptability of rumen epithelial cells to high-grain feeding [14]. Multi-omics studies, encompassing genomics, transcriptomics, proteomics, and metabolomics, have substantially contributed to our understanding of dairy cattle lactation. Significantly, multi-omics approaches have demonstrated their value in enhancing lactation performance in cows [15]. Integrating multi-omics data spanning various cellular functional levels provides insights into the underlying mechanisms of complex diseases, such as cancer. Multi-omics analysis offers distinct advantages in cancer research, as it allows for the utilization of multiple sets of biomarkers, offering greater specificity compared to single gene markers [16]. While there is currently no literature on using multi-omics analysis for studying cashmere fineness, the concept is feasible.

Following the experimental approach in published articles, a multi-omics analysis was employed to identify molecular markers linked to cashmere fineness [14–16]. Within cellular activities, minor alterations in macromolecular functions ultimately manifest at the metabolic level and become amplified in metabolites. Multi-omics analysis enables a more comprehensive comprehension of these processes. This study aimed to identify essential factors impacting cashmere fineness through integrating transcriptomics, translatomics, proteomics, and metabolomics. Analyzing key differential substances between coarse and fine cashmere skin tissues of LCG provides a more comprehensive understanding of the factors influencing cashmere fineness. Moreover, validating these factors through techniques like MRM and PRM will establish a scientific basis and introduce novel perspectives for future in-depth investigations into cashmere trait-related studies.

## Materials and methods

### Sample collection

Twelve Liaoning cashmere goats were utilized for the experiment, comprising six coarse cashmere type and six fine cashmere type goats selected from the Liaoning Province Modern Agricultural Production Base Construction Engineering Center in China. The mean cashmere diameter for the six coarse cashmere type Liaoning cashmere goats was 17.59 ± 0.31 μm (mean ± SD), with a range from 17.23 to 17.91 μm. The mean cashmere diameter for the six fine cashmere type Liaoning cashmere goats was 14.59 ± 0.21 μm (mean ± SD), with a range from 14.32 to 14.77 μm. The difference in cashmere diameters between the coarse and fine cashmere types was statistically significant (*P* < 0.01). The selected goats were not from the same group, had no familial relationship, and the sire and dam were unrelated for more than six generations, ensuring they were not inbred. These goats were 2-year-old ewes that received identical feeding management and were raised in the same growth environment. Skin samples were collected in May during the anagen phase of secondary hair follicles. First, the cashmere was removed, and then skin tissue of approximately 1 cm² was excised from the upper third of the right scapula of the Liaoning cashmere goats using surgical scissors and forceps. The tissue was promptly stored in liquid nitrogen. Local anesthesia (procaine) was administered to minimize animal discomfort during the skin tissue collection. The collected skin samples were utilized for four omics analyses.

### Methods of transcriptomics

Transcriptomics is sequenced using Short Read RNA-seq, a second-generation sequencing technology. The technical process primarily comprises RNA extraction and detection, library construction with quality control, sequencing, and bioinformatic analysis. The experimental procedure is the same as the “Transcriptomics analysis of cashmere fineness functional genes” [17].

### Methods of translatomics

Translatomics employs Ribo-seq sequencing technology. The specific method is to treat the ribosome-nascent peptide chain complex with a low concentration of RNase, degrade the RNA fragments without ribosome coverage, then remove the ribosomes, and finally use second-generation sequencing technology to detect the small fragments of RNA being translated that were protected by ribosomes. The experimental procedure is the same as the “Selection of Cashmere Fineness Functional Genes by Translatomics” [18].

### Methods of proteomics and PRM

Proteomics uses TMT technology. T The workflow, from tissue samples to the final data acquisition, encompasses essential stages, including protein extraction, quantification, detection, enzymatic cleavage, desalting, labeling, fraction separation, and mass spectrometry analysis. The experimental procedure is the same as the “Proteomic analysis of coarse and fine skin tissues of Liaoning cashmere goat” [19].

### Methods of metabolomics and MRM

Metabolomics relies on LC-MS technology, and the experimental procedure primarily encompasses metabolite extraction from samples, LC-MS/MS detection, and subsequent data analysis. The experimental procedure is the same as the “Metabolomic Analysis and MRM Verification of Coarse and Fine Skin Tissues of Liaoning Cashmere Goat” [13].

### Methods of association analysis

Correlation analyses between the omics data were conducted using Pearson statistics. Functional enrichment analyses were conducted based on pathways and terms shared across multiple omics datasets. A Padj value below 0.05 served as the threshold for determining significant enrichment. The software employed in the study is listed in Table 1.

**Table 1:** Software used for the analysis

Analytical projects	Omics	Software
Comparison analysis	Transcriptomics	Hisat2 (2.0.5)
	Translatomics	Tophat2 (2.0.12)
		Bowtie (1.0.1)
Quantitative analysis	Transcriptomics	FeatureCounts (1.5.0-p3)
		Stringtie (1.3.3b)
	Translatomics	FeatureCounts (1.5.0-p3)
		HTseq (v0.6.1)
	Proteomics	Proteome Discoverer (2.2)
	Metabolomics	Compound Discoverer (2.2)
Difference analysis	Transcriptomics	DESeq2 (1.16.1)
		EdgeR (3.18.1)
	Translatomics	DESeq2 (1.14.1)
		EdgeR (3.16.4)
	Proteomics	R (3.4.3)
	Metabolomics	R (3.4.3)
Differential TE gene analysis	Multi-omics	RiboDiff (V.0.2.1)
		Xtail (1.1.5)
Enrichment analysis	Multi-omics	ClusterProfiler (3.4.4)

## Results

### Identification information of transcriptomics, translatomics, proteomics, and metabolomics

In transcriptomics and translatomics, the sequencing error rate for individual base positions was under 1%, and the GC content ranged from 42 to 56% (Table S1, S2). The data from both omics datasets were sufficiently accurate for subsequent analysis. In proteomics, 3,999 proteins were quantified across all samples (Table S3). The PCA analysis reveals a notable distinction between the FT_LCG and CT_LCG groups (Fig. S1A). In metabolomics, a total of 625 metabolites were identified. In the PCA analysis, the quality control (QC) samples formed tight clusters, demonstrating excellent data reproducibility and affirming high data quality (Fig. S1B). All subsequent association analyses were conducted using these four omics datasets.

### Association analysis of transcriptomics and translatomics

#### Differential TE (translation efficiency) gene statistics

**Fig. 1:**
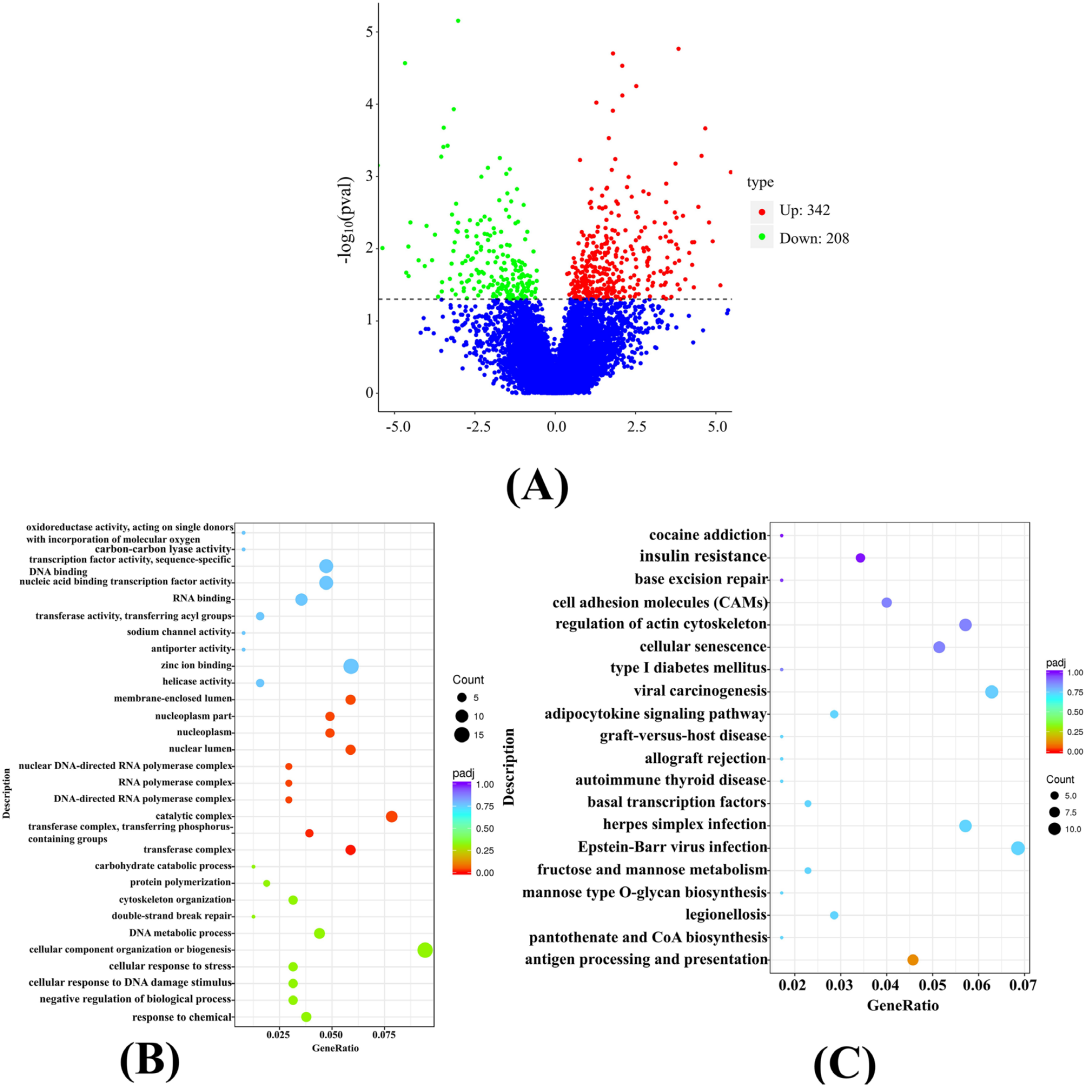
Transcriptomics and translatomics expression regulation diagrams. **(A)** Volcano Plot. Red indicates up-regulation, and green indicates down-regulation. **(B)** Bubble chart of GO enrichment analysis. The dot size reflects the number of genes annotated to the GO term, while the color gradient from red to purple indicates the degree of enrichment significance. **(C)** Bubble chart of KEGG enrichment analysis. Dot size corresponds to the number of genes annotated to the KEGG pathway, and the color gradient from red to purple signifies the level of enrichment significance

Modulation of translation efficiency is the primary mechanism governing gene translation levels, directly influencing protein production. 550 differentially regulated TE genes were identified, with 342 showing significant up-regulation and 208 exhibiting significant down-regulation. Details of the top 20 TE genes displaying significant differences are provided in Table S4. Furthermore, a volcano plot was employed to depict the differential gene expression between the FT_LCG and CT_LCG, as illustrated in Fig. 1A.

#### GO enrichment analysis

Through an analysis of the enrichment of differential TE genes, we could determine which biological functions of these genes were significantly linked to variations in cashmere fineness. The 30 GO terms displaying the most pronounced differences were visualized in a bubble chart within the GO enrichment analysis results (Fig. 1B). The findings revealed that the differential TE genes were significantly enriched in the transferase complex, the transferase complex, transferring phosphorus-containing groups, and the catalytic complex.

#### KEGG pathway enrichment analysis

The KEGG database is a comprehensive resource encompassing information related to genomic, chemical, and phylogenetic functions [20]. In the outcomes of the KEGG enrichment analysis, a bubble chart illustrating the 20 pathways exhibiting the most substantial differences was presented (Fig. 1C). The findings demonstrated that differential TE genes were significantly enriched in metabolic pathways such as Antigen processing and presentation, Antigen processing and presentation, and Legionellosis.

### Association analysis of transcriptomics and proteomics

#### Analysis of expression regulation

**Fig. 2:**
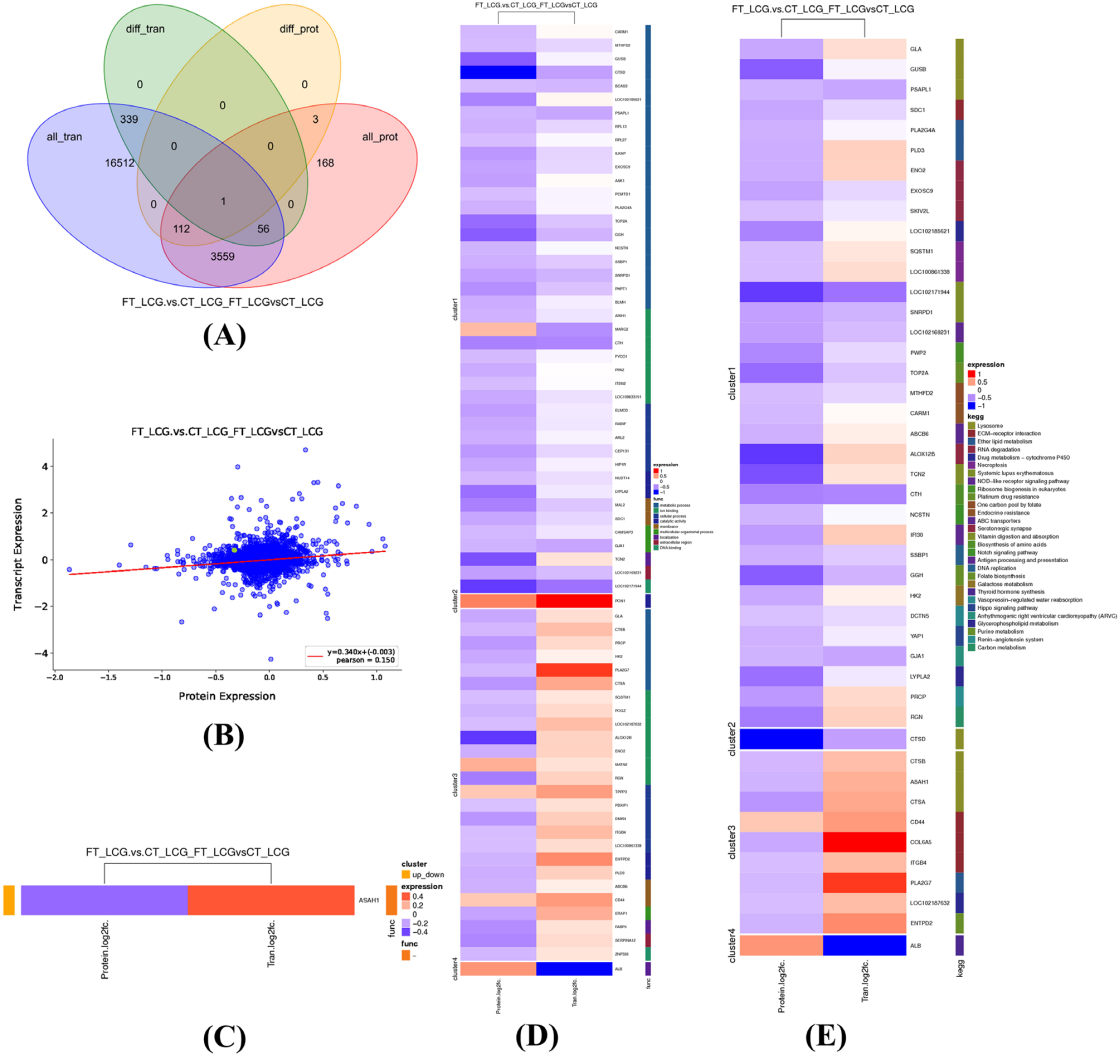
Transcriptomics and proteomics expression regulation diagrams. **(A)** Venn diagram of transcriptomic and proteomic expression regulation. In the figure, “all_tran” represents all genes obtained from the transcriptome, “diff_tran” represents differentially expressed genes identified by the transcriptome, “all_prot” represents all proteins identified by the proteome and “diff_prot” represents differential proteins identified by the proteome. **(B)** Scatter plot of expression correlation analysis. The green points represent proteins with a significantly different expression, and the blue points represent proteins with no significant difference in expression. **(C)** Clustering heat map of expression correlation analysis. **(D)** Clustering heat map of GO functional enrichment correlation analysis. **(E)** Clustering heat map of KEGG enrichment analysis. In **(C)**, **(D)**, and **(E)**, red indicates up-regulation, and blue indicates down-regulation

The mRNA data derived from transcriptomics was integrated with the protein data from proteomics. This analysis revealed an overlap in the genes (proteins) identified between the proteomics and transcriptomics datasets. The two omics datasets were jointly analyzed to determine correspondence. The outcomes were visually represented using a Venn diagram (Fig. 2A), which allowed for identifying common and unique genes (proteins) in different regions. The Venn diagram illustrates that were 3,559 genes identified in both transcriptomics and proteomics, with only one gene being commonly differential.

#### Expression association analysis

Genes identified in the transcriptomics and proteins identified in the proteomics were compared regarding their fold differences between the two omics datasets. A single co-expressed gene, *ASAH1*, exhibited significant differences and was identified (Fig. 2B). Plotting the above results as a heat map shows that the ASAH1 gene is down-regulated in proteomics but up-regulated in transcriptomics (Fig. 2C).

#### GO enrichment analysis

The GO enrichment results for proteins and genes from proteomics and transcriptomics were visualized as a heat map displaying the functional enrichment clusters. Enriched GO entries were grouped based on differential proteins. Among the GO functions, only catalytic activity was identified as up-regulated in both proteomics and transcriptomics. Four genes (*PON1, MATN2, TPPP3*, and *CD44*) exhibited up-regulation in both transcriptomics and proteomics (Fig. 2D), indicating a potential positive role in regulating cashmere fineness.

#### KEGG pathway enrichment analysis

KEGG pathway enrichment results for proteins and genes from both proteomics and transcriptomics were presented as a heat map displaying the clustering of enriched KEGG pathways. Enriched KEGG pathways were grouped based on differential proteins, and the outcomes are depicted in Fig. 2E. There were 12 KEGG pathways down-regulated in both the two omics and no KEGG pathways up-regulated in the two omics. Nevertheless, the *CD44* gene within the ECM receptor interaction pathway exhibited up-regulation in both omics, indicating a potential positive role in regulating cashmere fineness.

### Association analysis of transcriptomics and metabolomics

#### Association analysis of differential genes and differential metabolites expression

**Fig. 3:**
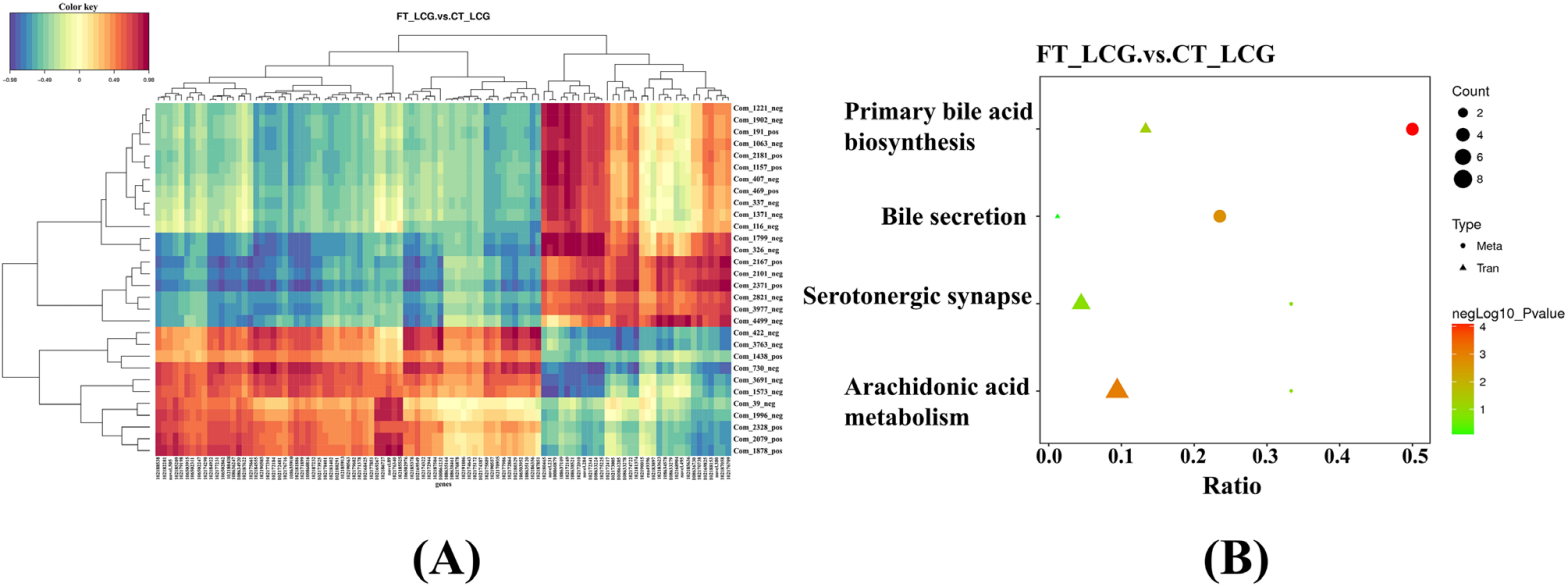
Transcriptomics and metabolomics expression regulation diagrams. **(A)** Heat map for association analysis of differential metabolites and genes expression. A redder color indicates a stronger positive correlation, while a stronger negative correlation is shown in bluer shades. **(B)** KEGG pathway association analysis of differential genes and differential metabolites

The correlation between genes and metabolites was assessed using Pearson correlation coefficients, considering the differential genes identified through transcriptomics and the differential metabolites filtered by metabolomics. A negative association is observed between a gene and a metabolite when the Pearson coefficient is below 0, while a positive association is indicated when the Pearson coefficient surpasses 0. The differential metabolites and genes were presented in ascending order of P values. The results showed that neodiosmin had the strongest positive correlation with the *MRPL17* gene, and N-Acetylsphingosine had the strongest negative correlation with the *ASS1* gene (Fig. 3A). Additionally, a strong negative correlation was observed between prostaglandin B2 and *ASS1*.

#### KEGG pathway enrichment analysis

Retrieve information on differential genes and metabolites from the KEGG Pathway Database and identify the metabolic pathways in which they are jointly involved [21]. To elucidate the critical biochemical and signal transduction pathways involved in differential metabolites and genes. Four KEGG pathways exhibited joint enrichment for differential genes and metabolites: arachidonic acid metabolism, bile secretion, primary bile acid biosynthesis, and serotonergic synapse (Fig. 3B). The arachidonic acid metabolic pathway showed the highest enrichment of differential genes. The majority of differential metabolites were enriched in two pathways: bile secretion and primary bile acid biosynthesis.

### Association analysis of proteomics and metabolomics

#### Association analysis of differential proteins and differential metabolites expression

**Fig. 4:**
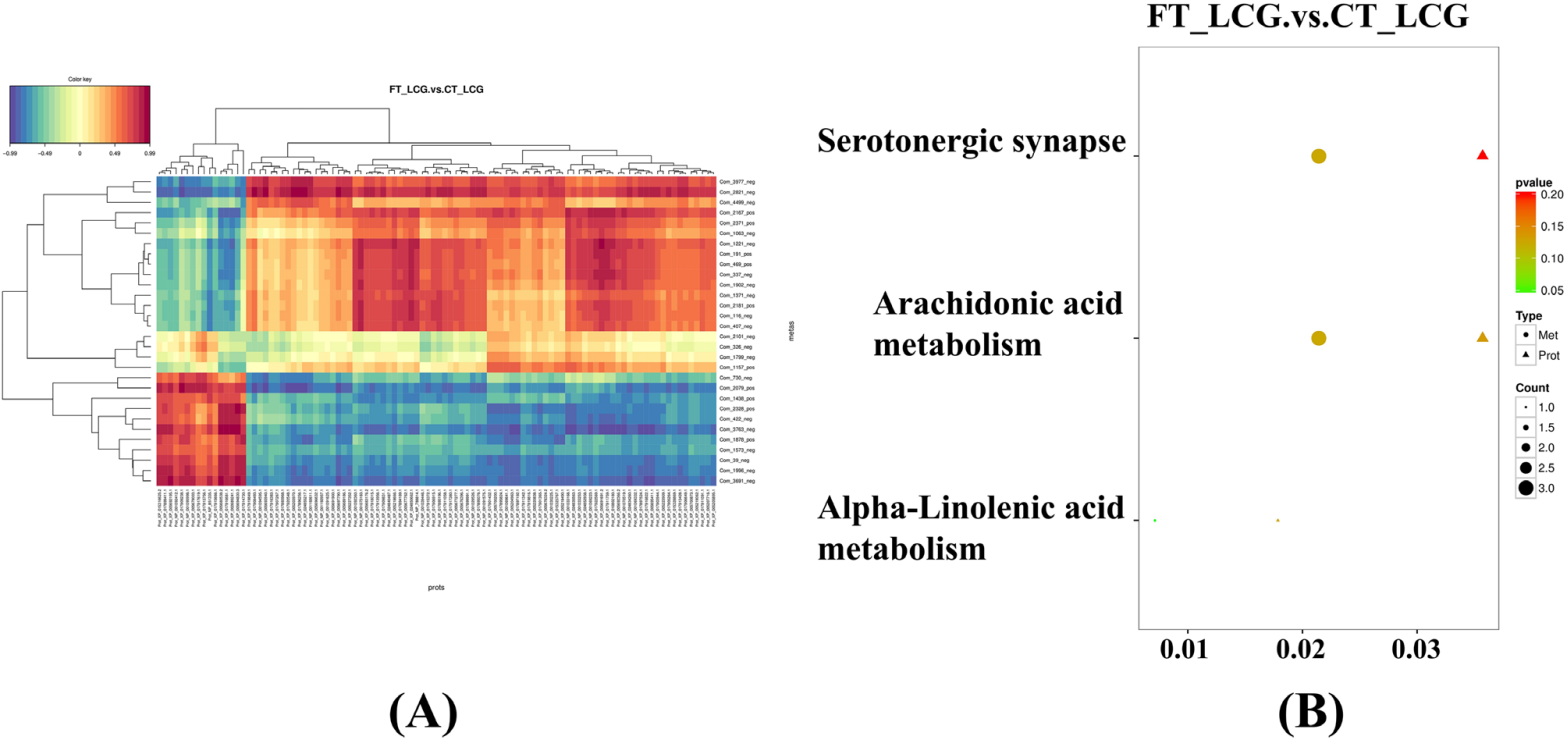
Proteomics and metabolomics expression regulation diagrams. **(A)** Heat map for association analysis of expression of differential proteins with differential metabolites. A redder color indicates a stronger positive correlation, while a stronger negative correlation is shown in bluer shades. **(B)** Association analysis of KEGG pathways for differential proteins with differential metabolites

Pearson correlation coefficients were employed to assess the extent of correlation between the differential proteins identified in proteomics and the differential metabolites identified through metabolomics. The correlation coefficient ranges from − 0.99 to + 0.99. A negative correlation exists between the protein and metabolite when the correlation coefficient is below 0, while a positive correlation is observed when the correlation coefficient surpasses 0. A correlation coefficient equal to 0 indicates no correlation. The expression trend was shown in Fig. 4A. The PE (18:1e/18:1) had the strongest positive correlation with LOC102179881. Prostaglandin B2 had the strongest negative correlation with TFAP2A.

#### KEGG pathway enrichment analysis

All differentially screened proteins and metabolites were collectively mapped in the KEGG pathway database to investigate their enrichment. The primary metabolic and signal transduction pathways were determined through which different proteins and metabolites interact. Three KEGG pathways, namely arachidonic acid metabolism, serotonergic synapse, and alpha-linolenic acid metabolism (Fig. 4B), exhibited enrichment of differential proteins and differential metabolites. The alpha-linolenic acid metabolism pathway showed the least significant enrichment, with the lowest number of enriched proteins and metabolites. In contrast, the serotonergic synapse pathway exhibited the highest significance.

### Association analysis of transcriptomics, translatomics, proteomics, and metabolomics

#### KEGG pathway enrichment analysis

**Fig. 5:**
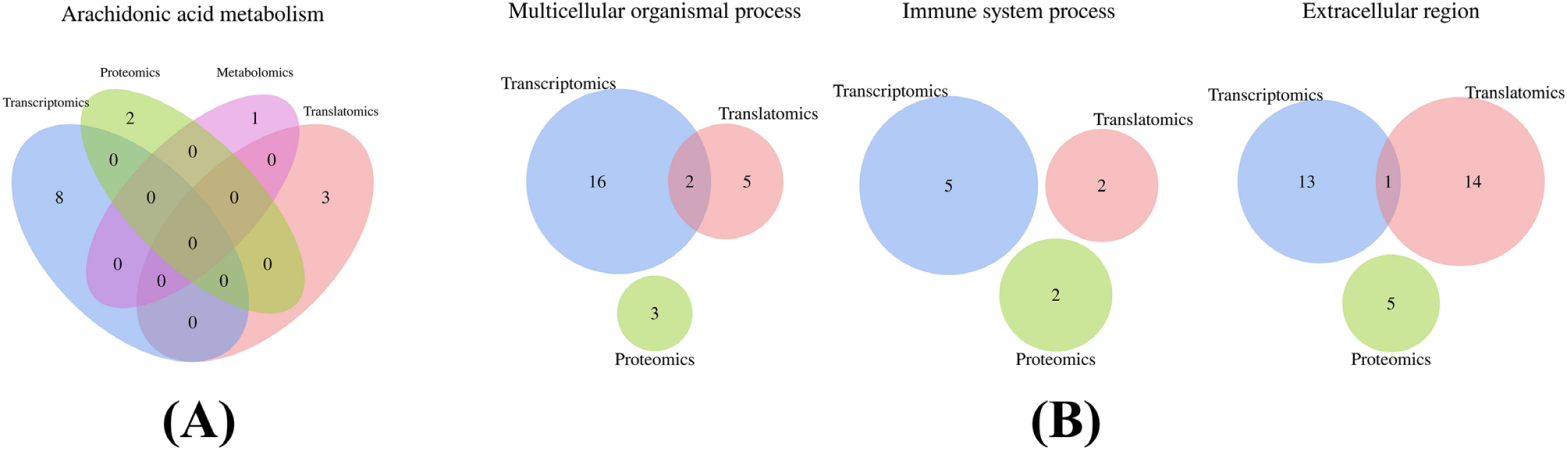
Transcriptomics, translatomics, proteomics, and metabolomics enrichment analysis. **(A)** Venn diagram of KEGG enrichment analysis. **(B)** Venn diagram of GO enrichment analysis

Arachidonic acid metabolism was the sole KEGG pathway that exhibited common enrichment across transcriptomics, translatomics, proteomics, and metabolomics. The Venn diagram (Fig. 5A) illustrates the quantitative relationships among the four omics. Detailed information regarding the genes, proteins, and metabolites enriched in each omics can be found in Table S5.

#### GO enrichment analysis

Enriched GO terms in transcriptomics, proteomics, and translatomics were collectively analyzed, with level 2 GO terms as a reference. Multicellular organismal process, immune system process, and extracellular region were the three GO terms enriched across all three omics. The Venn diagram (Fig. 5B) illustrates the quantitative relationships among the four omics. Detailed information about the specific genes and proteins can be found in Table S6. The results revealed that the gene *PLA2G12A* was co-enriched in both KEGG enrichment and GO enrichment analyses (Table S5, S6).

### PRM validation

**Fig. 6:**
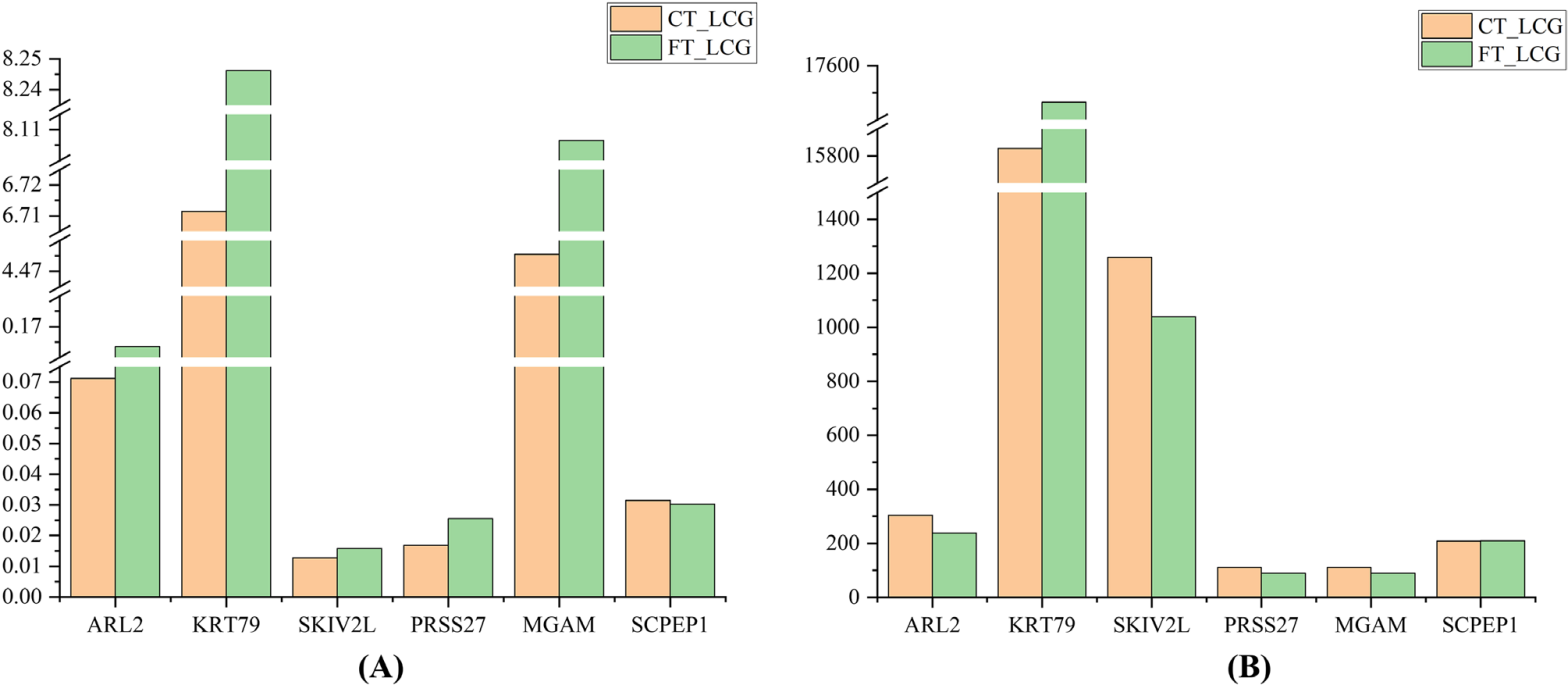
Trend plot of protein expression. **(A)** Target Protein PRM validation bar chart. **(B)** Bar chart of relative quantification of target proteins in TMT proteomics

We screened six candidate proteins in TMT proteomics. These six target proteins underwent analysis using PRM technology, and the relative expression differences of these target proteins between the two groups, FT_LCG and CT_LCG, were calculated. We compared the expression patterns of these six proteins in both PRM and TMT analyses (Fig. 6). The results demonstrated that the expression trends of KRT79 in PRM validation were in alignment with those observed in TMT proteomics and exhibited a positive influence on cashmere fineness.

### MRM validation

#### Screening of amino acids and their derivatives

**Fig. 7:**
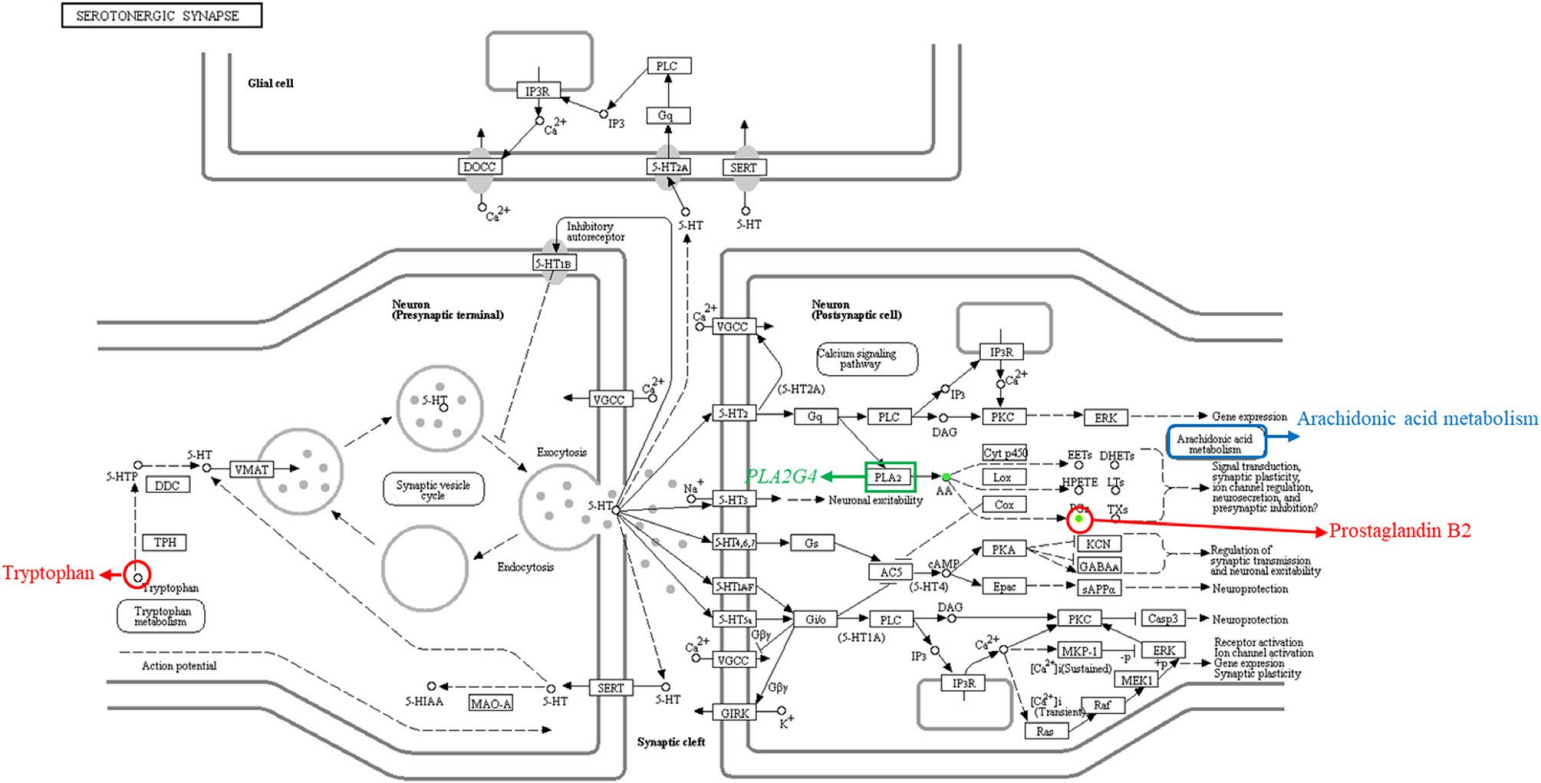
Serotonergic synapse pathway. Red circles indicate metabolites, green squares indicate genes, and blue squares indicate pathways

In the KEGG enrichment analysis [22], we observed that tryptophan, prostaglandin B2, and *PLA2G4* were enriched in the serotonergic synapse pathway (www.kegg.jp/kegg/kegg1.html), implying a coordinated biological role for these three substances. Furthermore, it appears that tryptophan may regulate prostaglandin B2 and *PLA2G4*. Additionally, the serotonergic synapse pathway exhibits interactions with the tryptophan metabolic pathway and the arachidonic acid metabolic pathway (Fig. 7). As a result, we conducted a quantitative analysis on 15 standard amino acids and their derivatives.

#### Trends in target metabolite expression

**Fig. 8:**
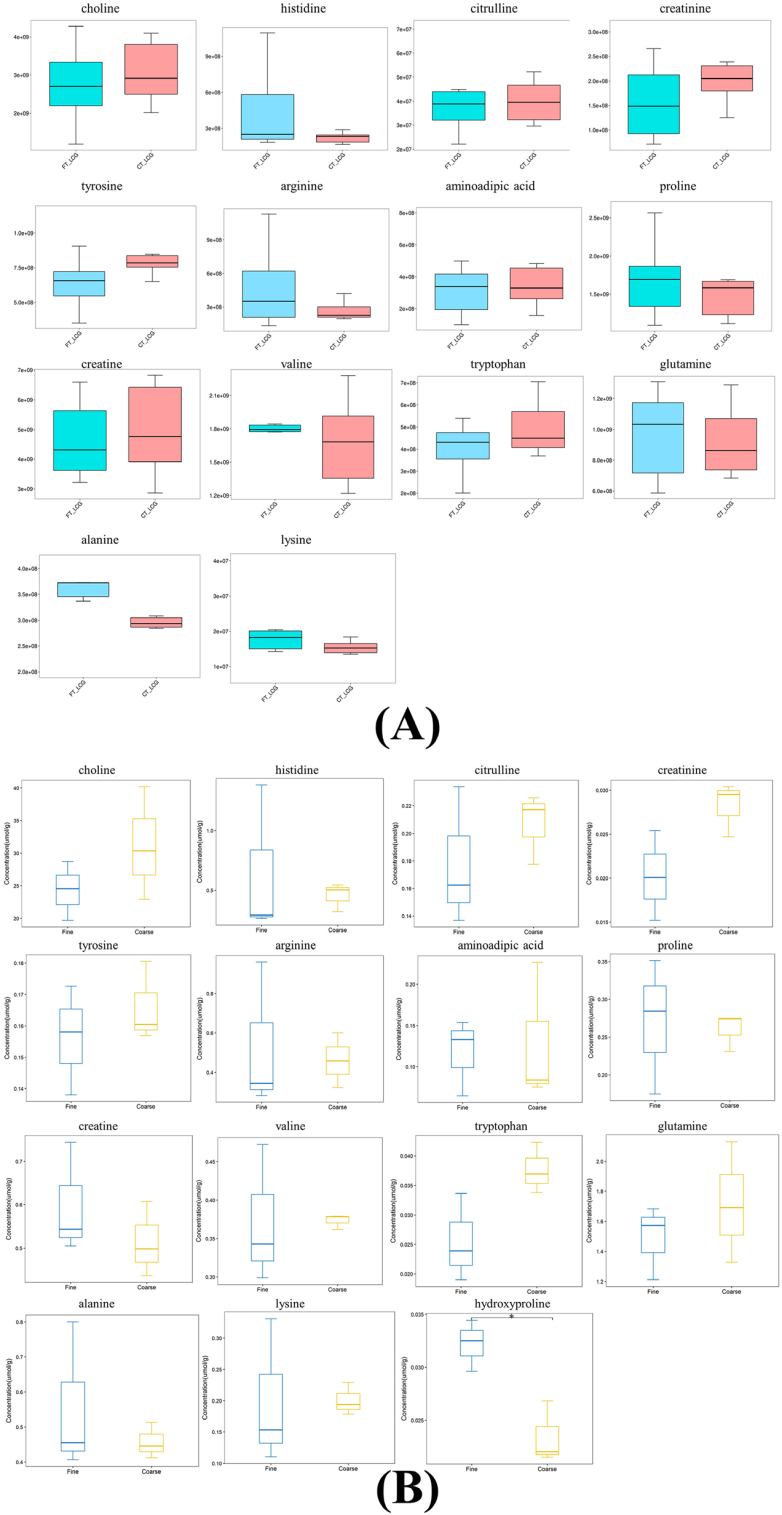
Trend plot of metabolite expression. **(A)** Box graph of amino acid expression trends in non-targeted metabolomics. Blue indicates FT_LCG, and red indicates CT_LCG. **(B)** Box graph of amino acid expression trends in MRM validation. Blue indicates FT_LCG, and yellow indicates CT_LCG. The * in the figure indicates 0.01 < *P* < 0.05, and those not labeled * indicate *P* > 0.05

We validated 15 amino acids and their derivatives using MRM techniques. The results indicated that 9 amino acids and their derivatives exhibited trends consistent with those observed in untargeted metabolomics. (Fig. 8). Additionally, we observed that hydroxyproline was undetected in non-targeted metabolomics but exhibited a significant difference in MRM (*P* < 0.05) and positively impacted cashmere fineness.

## Discussion

Multi-omics analysis has become a powerful tool with the widespread adoption of high-throughput technologies. These multi-omics approaches allow for integrating information from various omics levels, providing more robust evidence for biological mechanisms and facilitating the identification of key factors at a deeper level. In this study, we employed a combination of transcriptomics, translatomics, proteomics, and metabolomics to comprehensively analyze crucial substances within coarse and fine cashmere skin tissues, thereby enhancing our understanding of the regulators of cashmere fineness across multiple omics levels. Our integrated transcriptomic and translatomic analyses pinpointed *LOC102190399* (*PODNL1*) as the most significantly differentially expressed gene. While current research primarily focuses on *PODNL1’s* role in cancer development and progression, its potential influence on cell proliferation and differentiation is evident [23]. Studies have shown that knockdown and overexpression of *PODNL1* impact cell growth and migration [24]. Given that cashmere growth involves various processes of cell proliferation and differentiation within the skin, it is predicted that *PODNL1* may affect the cashmere growth process by influencing these cellular mechanisms. Furthermore, PODNL1 is associated with fibroblast-mediated wound healing and may regulate excessive extracellular matrix (ECM) deposition during the fibrotic response phase [25]. In our combined transcriptomic and proteomic analyses, we observed up-regulation of the *CD44* gene in both GO and KEGG clusters. *CD44* is involved in ECM receptor interactions, further supporting the potential involvement of *PODNL1* in regulating cashmere growth by influencing the extracellular matrix. Another common differential gene identified through the combined analysis of transcriptomics and proteomics was *ASAH1*. *ASAH1* encodes acidic ceramidase, a crucial player in maintaining the structure and function of the epidermis and a significant lipid in hair, affecting its physicochemical properties. Precise regulation of ceramide and free sphingosine base metabolism is vital for the homeostasis of hair follicle stem cells, the epidermis, and its appendages [26]. Additionally, unsaturated fatty acids can promote hair follicle regeneration and hair growth. This approach offers the advantages of minimal side effects, easy availability, and cost-effectiveness in treating hair loss, thus presenting promising prospects for development.

Following the integration of the four omics datasets, our analysis revealed that *PLA2G12A* and KRT79 play crucial roles in regulating cashmere fineness. *PLA2G12A* is particularly significant in phospholipid metabolism [27]. Phospholipase A2 (PLA2) is a vital metabolic and regulatory enzyme that catalyzes the hydrolysis of lipoproteins and glycerophospholipid molecules in cell membranes, resulting in the production of free fatty acids and lysolecithin [28]. Phospholipids play a crucial role in strengthening the epidermis and act as bioactive lipid mediators essential for maintaining homeostasis in the body. They serve as multifunctional phospholipid messengers through different signaling pathways within the skin. Additionally, lysophosphatidic acid is pivotal in various skin processes, including wound healing, skin barrier maintenance, skin hair growth, and hair follicle development [29]. KRT79, a member of the keratin family, assumes an essential role in shaping the hair follicle cavity [30]. In PRM validation, we observed higher expression of KRT79 in FT_LCG, consistent with the expression trend in TMT proteomics. These findings suggest that KRT79 may positively regulate the diameter of cashmere fibers while indicating that keratin’s expression is more stable. KRT79, classified as a type II keratin protein, influences capillary morphogenesis and regeneration in a newly migrating epithelial cell population. Its presence is critical for maintaining sebaceous stem cells in the skin, and its absence leads to abnormalities in the sebaceous glands and eyelid-lid glands [31]. Studies have shown that KRT79 expression may be a regulator of cell differentiation and cell motility. In the liver, KRT79 is controlled by PPARA and is highly correlated with liver injury, making it a potential diagnostic marker for human liver disease [32]. Keratin is an essential component of wool fibers, determining the structural characteristics of wool and cashmere. Genes encoding keratin are significant candidates in the hair follicle and hair studies [33]. Keratin can be divided into two main categories: keratin intermediate filament (KIF) and keratin-associated protein (KAP). KIF forms the essential structural backbone of hair, contributing to its stability. On the other hand, the content and structure of KAP can vary significantly among different species, suggesting its crucial role in regulating hair growth [10]. Although many studies have demonstrated the critical part of the keratin family in hair growth, the regulation of cashmere fineness by KRT79 needs further in-depth exploration.

The integration of the four omics datasets unveiled the consistent enrichment of the arachidonic acid metabolism pathway in all four of them. Arachidonic acid is crucial for overall metabolism, particularly in the skin, and when it is supplied adequately, it contributes to shiny and well-hydrated hair. When arachidonic acid is acted upon by cyclooxygenase, it converts into prostaglandin intermediate metabolites, which are further synthesized into various prostaglandins with diverse biological activities by different prostaglandin synthetases. In this study, we focused on enriching the arachidonic acid metabolic pathway with a specific metabolite, prostaglandin B2, which appears to be a pivotal metabolite in regulating cashmere fineness. Prostaglandins are unsaturated fatty acids distributed in various body tissues, playing essential roles in cell proliferation, differentiation, and apoptosis. The complex mechanism of prostaglandin metabolism in hair cells centers primarily around the hair papilla epithelium, where prostaglandin E2 (PGE2) and prostaglandin F2α (PGF2α) occur. This suggests that prostaglandins are likely involved in hair growth and hair follicle differentiation [34]. While PGE2 and PGF2α promote hair growth within the follicle, prostaglandin D2 (PGD2) restricts hair growth. Prostaglandin derivatives have been found to enhance the transition of hair follicles from telogen to anagen, leading to increased hair count, density, and length [35]. Additionally, prostaglandins may play a role in the development of androgenetic alopecia. Clinical trials have demonstrated that topical application of 0.1% latanoprost effectively improves hair density in patients with androgenetic alopecia [36]. The antagonism between Latanoprost and PGF2α receptors has been shown to influence the follicle growth cycle and hair growth [37]. Latanoprost has been approved to enhance local circulation to promote eyebrow and eyelash growth. These findings suggest that prostaglandins and their receptors could potentially serve as targets for treating androgenetic alopecia and may also be related to the growth and development of cashmere in LCG.

## Conclusion

In this study, we discovered that the substances influencing cashmere fineness were primarily enriched in multicellular organismal process, immune system process, and extracellular region. These substances were also associated with arachidonic acid metabolism. Additionally, the study identified *PLA2G12A*, KRT79, and prostaglandin B2 as pivotal factors that regulate cashmere fineness.

### Electronic supplementary material

Below is the link to the electronic supplementary material.

Supplementary Material 1

## Data Availability

The data supporting this study’s findings are available from the corresponding author upon reasonable request.

## List of abbreviations

LCG: Liaoning cashmere goat
FT_LCG: Fine cashmere type Liaoning cashmere goats
CT_LCG: Coarse cashmere type Liaoning cashmere goats
PRM: Parallel reaction monitoring
MRM: Multiple reaction monitoring
Padj: P adjust
GO: Gene Ontology
KEGG: Kyoto Encyclopedia of Genes and Genomes
ECM: Extracellular matrix
TMT: Tandem Mass Tag
TE: Translation Efficiency
