# Supplementary material for: Multi-omics analysis of functional substances and expression verification in cashmere fineness
Source: BMC Genomics. 2023 Nov 28;24:720. doi: 10.1186/s12864-023-09825-0 (PMC10685610; doi:10.1186/s12864-023-09825-0)
Supplement: Supplementary file 1 — Supplementary Material 1 [file 12864_2023_9825_MOESM1_ESM.docx]

| Sample | Raw reads | Clean reads | Error rate | Q20 | Q30 | GC content |
| --- | --- | --- | --- | --- | --- | --- |
| FT_LCG_1 | 46,321,014 | 44,024,988 | 0.02% | 98.11% | 94.56% | 51.46% |
| FT_LCG_2 | 46,730,722 | 43,668,406 | 0.02% | 98.19% | 94.78% | 50.85% |
| FT_LCG_3 | 48,040,536 | 44,506,336 | 0.02% | 98.42% | 95.33% | 52.12% |
| CT_LCG_4 | 46,503,644 | 43,080,972 | 0.02% | 98.38% | 95.21% | 51.51% |
| CT_LCG_5 | 45,609,522 | 43,605,410 | 0.02% | 98.45% | 95.30% | 51.33% |
| CT_LCG_6 | 45,602,548 | 43,037,778 | 0.02% | 98.30% | 95.08% | 51.89% |

**Table S1** Sample sequencing data quality in transcriptomics

**Table S2** Sample sequencing data quality in translatomics

| Sample | Raw reads | Error rate | Q20 | Q30 | GC content |
| --- | --- | --- | --- | --- | --- |
| FT_LCG_1 | 50,666,392 | 0.01% | 99.10% | 96.97% | 55.41% |
| FT_LCG_2 | 50,286,756 | 0.01% | 99.12% | 96.94% | 55.09% |
| FT_LCG_3 | 52,749,309 | 0.01% | 98.97% | 96.79% | 55.85% |
| CT_LCG_4 | 49,894,812 | 0.01% | 98.81% | 96.54% | 55.57% |
| CT_LCG_5 | 51,599,006 | 0.01% | 99.13% | 97.09% | 55.29% |
| CT_LCG_6 | 59,901,380 | 0.01% | 99.15% | 97.13% | 55.94% |

**Table S3** Proteomics identification overview

| Total spectra | Matched spectrum | Peptide | Identified protein | ALL |
| --- | --- | --- | --- | --- |
| 314,881 | 70,066 | 29,446 | 4,022 | 3,999 |

**Table S4** Top 20 differentially expressed TE genes

| Gene id | Gene Name | log_2_FoldChange | P value | P.adjust (Padj) |
| --- | --- | --- | --- | --- |
| 102190399 | *LOC102190399* | -6.005187657 | 1.10E-05 | 0.061492 |
| 102178021 | *ITIH4* | 3.837280025 | 1.71E-05 | 0.061492 |
| 102175337 | *FRMD4B* | 1.798622283 | 1.98E-05 | 0.061492 |
| 102184185 | *CCNE1* | 2.086546502 | 2.94E-05 | 0.061492 |
| 102188389 | *DENND1C* | 2.521558221 | 5.62E-05 | 0.100772 |
| 102191129 | *SHANK3* | 2.08914259 | 7.59E-05 | 0.119159 |
| 102172989 | *CHAF1A* | 1.280095525 | 9.52E-05 | 0.132785 |
| 102175721 | *TOPAZ1* | -3.156989891 | 0.000117 | 0.140578 |
| 102190475 | *FAM65B* | 1.793876951 | 0.000123 | 0.140578 |
| 102172352 | *JAKMIP2* | -3.469457163 | 0.000212 | 0.194457 |
| 102173569 | *LOC102173569* | 4.669837843 | 0.000217 | 0.194457 |
| 102179737 | *PRKAG1* | 1.668625301 | 0.000295 | 0.246842 |
| 102172524 | *NAP1L5* | -3.348768078 | 0.000375 | 0.28844 |
| 102191254 | *COL22A1* | -3.479799607 | 0.000391 | 0.28844 |
| 106501932 | *NHLH2* | -7.303833401 | 0.000464 | 0.32389 |
| 102188917 | *FAM185A* | 4.554038023 | 0.000521 | 0.324215 |
| 102183081 | *MED30* | -3.546948715 | 0.000534 | 0.324215 |
| 102177314 | *NOL12* | -1.723668369 | 0.000555 | 0.324215 |
| 102181106 | *LOC102181106* | 1.869466716 | 0.000576 | 0.324215 |
| 106502488 | *SCAF8* | 0.771709994 | 0.000594 | 0.324215 |

**Table S5** KEGG pathway common to transcriptomics, translatomics, proteomics, and metabolomics

| KEGG pathway | Transcriptomics | Translatomics | Proteomics | Metabolomics |
| --- | --- | --- | --- | --- |
| Arachidonic acid metabolism | *LOC108634920/LOC102176340/ALOX5/LOC102187504/LOC102184867/GPX2/TBXAS1/LOC102169702* | *LOC102190498/PTGES/PLA2G12A* | PLA2G4A/ALOX12B | Prostaglandin B2 |

**Table S6** Common GO terms enriched by transcriptomics, translatomics, and proteomics

| GO_Term | Transcriptomics | Translatomics | Proteomics |
| --- | --- | --- | --- |
| multicellular organismal process | *TNF/LOC102186727/CXCL9/PRG4/LOC106503915/IL1B/CD74/LOC102187998/CXCL10/LOC102189356/CD40LG/LOC106503481/CCL16/GCSAML/CCL27/LOC102180664/LOC108633459/LOC102188267* | *LOC102181854/LOC102181202/LST1/TMEM173/LOC106503915/PRG4/LOC102182395* | CAMSAP3/GJA1/ERAP1 |
| immune system process | *TMEM119/MYF6/EDN3/C14H8orf22/WNT16* | *DKK2/TMEM173* | C9/ERAP1 |
| extracellular region | *IGFBP2/CXCL9/IL1B/CXCL10/EDN3/MMP9/IGFBPL1/CCL16/WNT16/LOC100860781/CCL27/THBS4/CSN3/LOC102168428* | *LOC102173761/LOC102181854/PLAT/CSN2/C3/THBS4/IGFBP4/DKK2/THBS2/LOC102182395/MMP19/FN1/WFDC1/**PLA2G12A/COL15A1* | ALB/PON1/SERPINA12/KRT79/LOC102169231 |

**
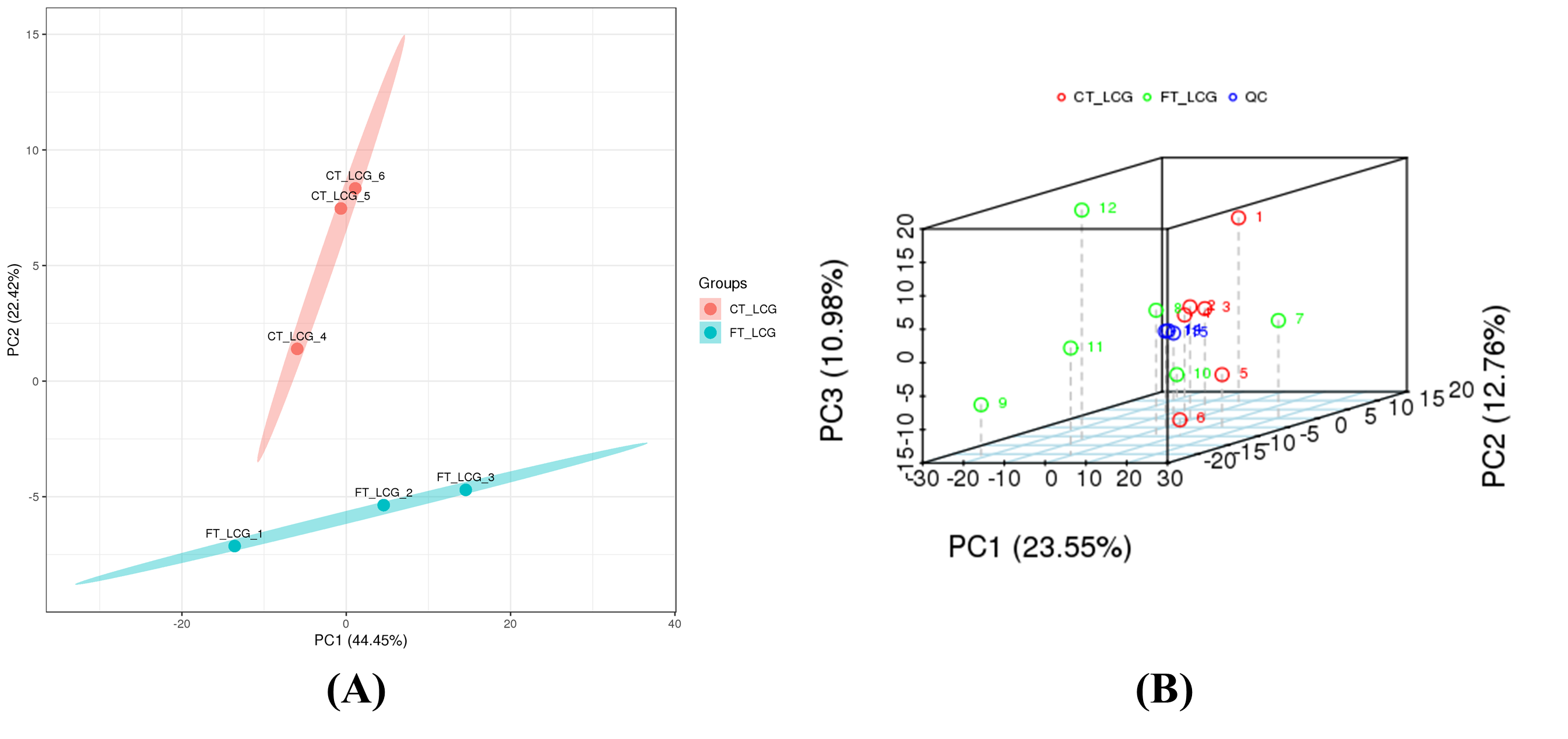
**

**Fig. S1** PCA analysis. (A) PCA analysis of proteomics. Blue indicates FT_LCG and red indicates CT_LCG. (B) PCA analysis of metabolomics. Green indicates FT_LCG, red indicates CT_LCG, and blue indicates QC.
